# Structural and functional retinal changes in patients with type 2 diabetes without diabetic retinopathy

**DOI:** 10.1080/07853890.2022.2095010

**Published:** 2022-07-04

**Authors:** Qiannan Chai, Yimin Yao, Congrong Guo, Hong Lu, Jingxue Ma

**Affiliations:** Department of Ophthalmology, The Second Hospital of Hebei Medical University, Shijiazhuang, China

**Keywords:** Diabetes, type 2, diabetic retinopathy, optical coherence tomography, microperimetry, optical coherence tomographic angiography

## Abstract

**Objective:**

The characteristics of the early changes in preclinical diabetic retinopathy (DR) are poorly known. This study aimed to analyse the changes in the structure and function of the fundus in diabetic patients without diabetic retinopathy (NDR).

**Methods:**

This prospective study enrolled patients with type 2 diabetes and healthy controls from April to December 2020. Retinal sensitivity was measured by microperimetry. The peripapillary retinal nerve fibre layer (p-RNFL) thickness, macular retinal thickness, and retinal volume were measured by optical coherence tomography (OCT). The vessel density (VD) and perfusion density (PD) of the peripapillary area, as well as the foveal avascular zone (FAZ) area, FAZ perimeter, and FAZ circularity, were measured by optical coherence tomographic angiography (OCTA).

**Results:**

A total of 71 cases (100 eyes) were enrolled in the study, including 34 cases (51 eyes) in the NDR group and 37 cases (49 eyes) in the control group. The mean retinal sensitivity was lower in the NDR group than in the control group for all sectors (all *p* < .001). Compared with controls, the NDR group showed thinner p-RNFL in the T sector (76.24 ± 14.29 vs. 85.47 ± 19.66 µm, *p* = .035). The NDR group had a thinner retina in the N2 sector (304.55 ± 16.07 vs. 312.02 ± 12.30 µm, *p* = .010). The PD of DCP was lower in the N2 sector in the NDR group (44.92 ± 11.77 vs. 50.27 ± 6.37%, *p* = .044). The VD was higher in the NDR group in RPCP-S/N/I, and the PD was higher in the RPCP-S/N (all *p* < .05). The frequencies of perifoveal capillary drop-out, notched or punched out borders of the superficial FAZ, and loss of smooth contour were all higher in the NDR group (all *p* < .05).

**Conclusion:**

The structure (p-RNFL thickness, VD, and PD) and function (retinal sensitivity) display some changes in diabetic patients even if they had not been found to have DR.

**Key messages**
1. Decreased retinal sensitivity was observed in diabetic patients before the onset of diabetic retinopathy.
2. Compared with the control group, we found the changes in vessel density or perfusion density in a certain area, whether in SCP, DCP, or RPCP in the NDR group.
3. Before the onset of diabetic retinopathy, the structure and function of the retina in diabetic patients had changed.

## Introduction

Chronic hyperglycaemia (and other factors) can trigger biochemical and physiological changes, resulting in microvascular damage and retinal dysfunction [1]. Diabetic retinopathy (DR) is a progressive complication of diabetes in which retinal vascular damage and abnormalities can lead to vision impairment and blindness [1]. The prevalence of DR is 34.6% among adults with diabetes [2]. Risk factors of DR include long-duration diabetes, chronic hyperglycaemia, nephropathy, hypertension, and dyslipidemia [3]. DR can be classified based on disease severity: nonproliferative DR characterised by retinal vascular abnormalities, proliferative DR characterised by retinal neovascularization, vascular abnormalities, and diabetic macular edema characterised by thickening of the retina near the macula [3]. Regular screening and diabetes control can help reduce the risk of developing DR [1,4].

Hyperglycaemia and associated altered metabolic pathways can lead to neural damage (neurodegeneration),vascular damage, and impaired neurovascular unit function [3]. Neurodegeneration, leading to fewer retinal ganglion cells and thinner nerve fibre layers, can be caused by increased extracellular glutamate accumulation induced excitotoxicity and reduced retinal production of neurotrophins [3]. Vascular damage can be caused by increased vascular endothelial growth factors and the production of erythropoietin and inflammatory mediators [3]. The impaired neurovascular unit function can impair the ability of the retina to regulate local blood flow in response to neural activity and metabolic demands [3]. Since chronic hyperglycaemia can induce retinal changes even before the onset of type 2 diabetes [1], some studies reported that neural structural changes in the retina could be observed in patients with early-stage type 2 diabetes [5,6].

A recent study suggested that about 40% of diabetic patients without DR had microvascular abnormalities according to optical coherence tomographic angiography (OCTA) [5]. Previous studies reported either a decrease in the retinal nerve fibre layer (RNFL) thickness in diabetic patients [7,8] or no significant changes [9–14]. Evidence suggests that microvascular changes and neuronal impairment can occur in early DR [15,16]. A study highlighted that the retinal changes might be localised in early DR instead of affecting the entire retina [17]. In addition, microperimetric sensitivity in diabetic patients, either with or without DR, was reported to be reduced significantly compared with non-diabetic control subjects [18,19]. It is also reported that parafoveal and perifoveal vessel density (VD) of superficial capillary plexus (SCP) and deep capillary plexus (DCP) is decreased in diabetic patients without clinically detectable retinopathy in comparison to healthy controls [20]. Still, the characteristics of the early changes in preclinical DR are poorly known.

Therefore, this study aimed to analyse the relationship between early retinal structural changes and functional impairment in early-stage diabetic patients. The results help the understanding and the management of DR.

## Patients and methods

### Patients

This prospective study enrolled patients with type 2 diabetes admitted to the Department of Endocrinology and the Department of Ophthalmology of the Second Hospital of Hebei Medical University from April to December 2020. This study was approved by the Ethics Committee of the Second Hospital of Hebei Medical University (approval number: 2020-R146). All participants signed the informed consent form. The study was carried out according to the tenets of the Declaration of Helsinki and the Good Clinical Practices.

The inclusion criteria were (1) 30–70 years old, (2) diagnosed with type 2 diabetes, (3) and no diabetic retinopathy (NDR) was found by ophthalmoscope and fundus fluorescein angiography [21].

A group of healthy individuals who underwent routine health check-ups during the same period was included and matched 1:1 for age with the patients. The control group included healthy adults without systemic diseases. The inclusion criteria for the control group were (1) 30–70 years old and (2) indirect ophthalmoscope examination after mydriasis showed no lesions on the fundus of both eyes.

The exclusion criteria for both groups were (1) dioptre ≥ +3.00 D or ≤-3.00 D, (2) history of retinal vein occlusion, age-related macular degeneration, epimacular membrane, retinal vasculitis, uveitis, glaucoma, or other eye diseases, (3) history of eye surgery or laser surgery, including vitrectomy, transpupillary thermotherapy, intravitreal drug injection, macular laser photocoagulation, or phacoemulsification and intraocular lens implantation within 6 months, (4) with poor fixation behaviour or severe refractive interstitial opacity that affects the fundus examination, (5) treated with glucocorticoids, (6) systemic diseases including hematological diseases, cardiac insufficiency, coronary heart disease, systemic lupus erythematosus, and anaemia; or (7) fail to obtain qualified images, i.e. with a signal strength index (SSI) <6 out of 10. The form and density of cataracts highly influence macular sensitivity measurements [22]. The severe opacity of cataracts will lead to a decrease in sensitivity. Therefore, significant cataracts (more than N2 C1 P1) according to LOCS III standard photographs (LOCS III; LOCS chart III; Leo T Chylack, Harvard Medical School, Boston, MA, USA) were excluded.

### Microperimetry

**Figure 1.**
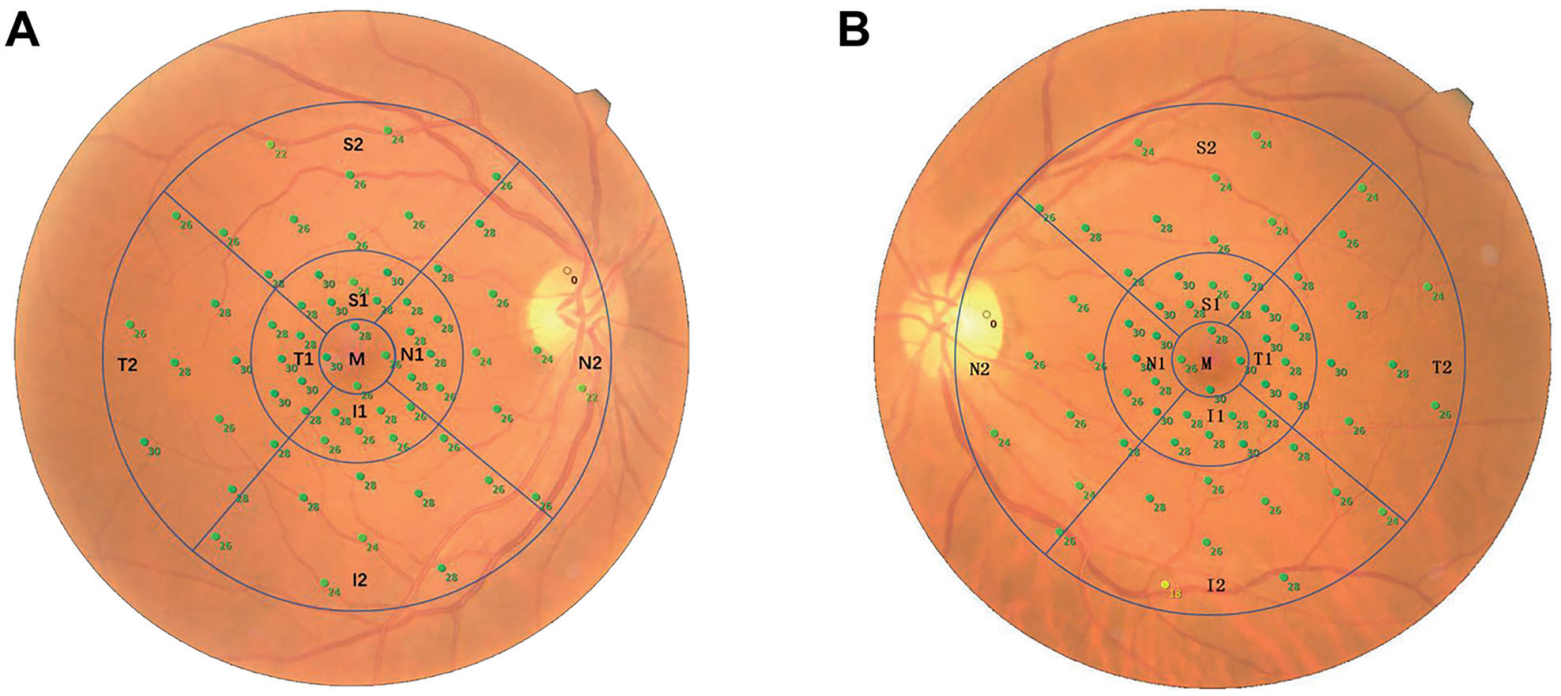
Diagram of the ETDRS chart. The retinal sensitivity test results of microperimetry are automatically matched with the fundus photos. (A) Right eye; (B) Left eye.

A microperimeter (MP-3, Nidek Technologies, Aichi, Japan) was used to measure the retinal sensitivity. All patients were treated with compound tropicamide eye drops for pupil dilation 30 min before the examination. Then the examination was carried out in a dark room with the opposite eye covered. All subjects were fully informed of the precautions before the examination and were tested with five stimulating light spots before the formal examination to confirm that the subjects could effectively cooperate with the examination. A white background was set, the brightness of the background light was 31.4 asb, and the brightness of the maximum stimulus was 10,000 asb. A Goldmann III stimulation light source was selected, with a 4-2 threshold strategy. The initial stimulus intensity of the cursor was 20 dB, the dynamic range of the stimulus was 0–34 dB, and the time interval between the visual target appearance was 200 ms. The participants fixated on a red cross with a diameter of 1°. A program of 64 test points was adopted, and the test range was 30° in the macular area. After the retinal sensitivity test was completed, a colour photo of the fundus was taken. The microperimeter automatically matched the sensitivity test results of microperimetry with the fundus photos. During the examination process, the automatic eye-tracking system tracked the retina in real-time to ensure that each stimulus reached the set retinal location. The NAVIS-EX analysis software (version 1.8.0) built in the microperimetry machine was used for analysis. Based on the Early Treatment of Diabetic Retinopathy Study (ETDRS) chart, the 64 test points were divided into nine sectors, which were the middle (M) subfield, the superior (S1), temporal (T1), inferior (I1), and nasal (N1) quadrants of the inner ring, and the superior (S2), temporal (T2), inferior (I2), and nasal (N2) quadrants of the outer ring. The diameters of the three circles were 1, 3, and 6 mm, respectively (Figure 1). The mean retinal sensitivity of these nine sectors and the superior (S1 + S2), temporal (T1 + T2), nasal (N1 + N2), and inferior (I1 + I2) quadrants were measured. Notably, when calculating mean retinal sensitivity in the N2 quadrant, light spots that fall in the optic disc region were excluded.

### Optical coherence tomography (OCT) measurement

OCT examination was performed using a Nidek RS-3000 Advance 2 Angioscan (Nidek Technologies, Aichi, Japan) by a single trained examiner with a scan density of 256 A-scans (horizontal) × 256 B-scans (vertical). Both eyes in all patients were scanned. The four-quadrant (temporal, superior, nasal, and inferior) peripapillary retinal nerve fibre layer (p-RNFL) thickness was measured by the built-in software after automatically positioning a 3.45 mm diameter circle at the centre of the optical disc. The macular area of 6 mm × 6 mm was scanned to measure the retinal thickness and retinal volume of the above-mentioned nine sectors.

### OCTA measurement

**Figure 2.**
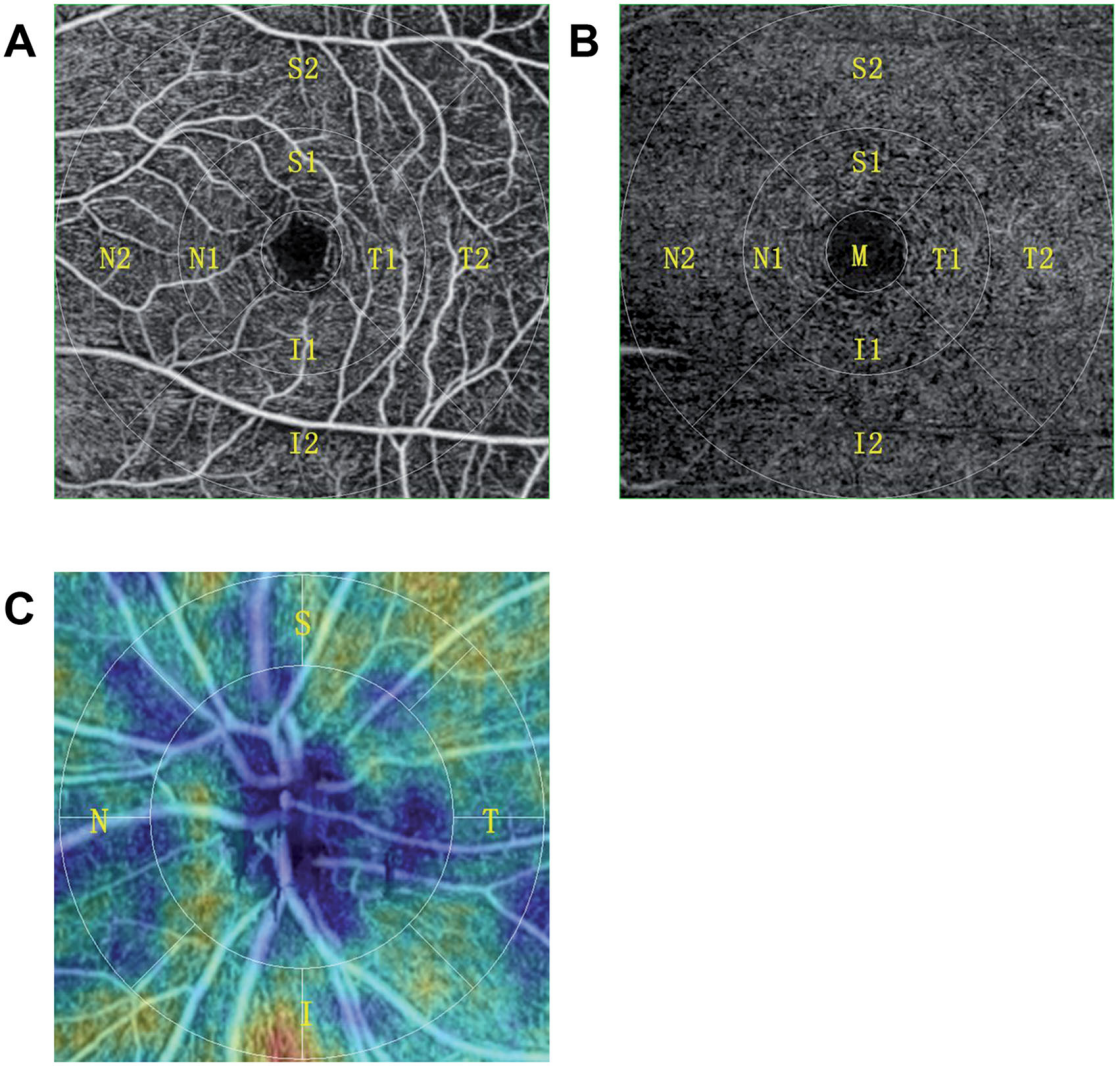
OCTA scans. (A) The superficial capillary plexus; (B) deep capillary plexus; (C) radial peripapillary capillary plexus.

OCTA examination was performed using the Nidek RS-3000 Advance 2 Angioscan (Figure 2). The four-quadrant (temporal, superior, nasal, and inferior) radial peripapillary capillary plexus (RPCP) of the optic disc was scanned in an area of 4.5 mm× 4.5 mm, while SCP and DCP of the macular region were scanned in an area of 6 mm× 6 mm based on the ETDRS chart. Sectorial VD and perfusion density (PD) of the RPCP, SCP, and DCP were measured. The foveal avascular zone (FAZ) area, FAZ perimeter, and FAZ circularity of the SCP were measured by 3 × 3 mm scans.

### Data collection and definitions

Age, sex, and body mass index (BMI) were collected. The blood pressure was measured using an electronic sphygmomanometer (Yuwell, Yuyue Medical Equipment & Supply Co., Ltd., Jiangsu, China) and measured three times to calculate the mean value. Fasting blood (5 mL) was obtained from the median cubital vein to examine the levels of triglycerides (TG), total cholesterol (TC), high-density lipoprotein cholesterol (HDL), low-density lipoprotein cholesterol (LDL), blood urea, and creatinine. The duration of diabetes was recorded, and the level of HbA1c was measured in the NDR group. The participants’ best-corrected visual acuity (BCVA) and intraocular pressure (IOP) were measured.

### Statistical analysis

SPSS 22.0 (IBM, Armonk, NY, USA) was used for analysis. The Shapiro–Wilk test was used to test the normality of the continuous variables. Those consistent with the normal distribution were presented as means ± SD and analysed using Student’s *t*-test; those that did not conform to the normal distribution were represented as median (P25, P75) and analysed using the Mann–Whitney *U*-test. Categorical variables were presented as *n* (%) and analysed using the chi-square test or Fisher test. The Pearson correlation analysis was used for two variables with a normal distribution, while the Spearman correlation analysis was used if at least one of the two variables did not conform to the normal distribution. The *p*-values were adjusted for multiple comparisons using the Holm–Bonferroni method. *P*-values <.05 were considered statistically significant.

The post hoc power analysis indicated that with a sample size of 34 patients per group (two groups, for a total sample size of 68 patients), it was expected to achieve at least 80% power to detect a difference of 2% between the null hypothesis (both groups mean values were 4.2%) and the alternative hypothesis (the mean in the NDR group was 1.07% with estimated group SD of 7.5% and 11.0%) and with a significance level (alpha) of 0.05 using a 2-sided 2-sample *t*-test.

## Results

### Characteristics of the participants

After excluding patients and eyes based on the exclusion criteria, 71 patients (100 eyes) were included in this study, including 34 patients (51 eyes) in the NDR group and 37 patients (49 eyes) in the control group (Supplement Table 1). In the control group, 12 healthy people had two eyes included; in the NDR group, the eyes of 17 patients met the eligibility criteria. The demographic characteristics and laboratory test results are presented in Table 1. There were no differences in all the characteristics between the two groups (all *p* > .05).

**Table 1.** Characteristics of the participants.

Characteristics	NDR (*n* = 34)	Control (*n* = 37)	*p*
Sex, *n* (%)			.119
Male	21 (61.8%)	16 (43.2%)	
Female	13 (38.2%)	21 (56.8%)	
Age (years), median (P25, P75)	54.5 (50, 62)	53 (48, 57)	.302
BMI (kg/m^2^), mean ± SD	25.2 ± 2.4	24.6 ± 2.0	.235
SBP (mmHg), mean ± SD	124 ± 11	121 ± 9	.249
DBP (mmHg), mean ± SD	77 ± 8	76 ± 6	.521
TG (mmol/L), median (P25, P75)	1.44 (1.01, 2.2)	1.23 (0.98, 1.67)	.236
TC (mmol/L), mean ± SD	4.41 ± 0.820	4.37 ± 0.562	.802
HDL (mmol/L), mean ± SD	1.41 ± 0.270	1.43 ± 0.241	.816
LDL (mmol/L), mean ± SD	2.72 ± 0.719	2.82 ± 0.416	.481
Serum urea (mmol/L), median (P25, P75)	5.05 (4.5, 5.9)	5.02 (4.74, 5.36)	.791
Serum creatinine (μmol/L)	64.81 ± 11.005	68.34 ± 8.674	.136
BCVA (LogMAR), median (P25, P75)	0.00 (0.00, 0.10)	0.00 (0.00, 0.00)	.138
IOP (mmHg), mean ± SD	16.07 ± 2.04	15.38 ± 2.06	.102
Duration of diabetes (years)	8.6 ± 5.38	–	–
HbA1c (%), median (P25, P75)	7.05 (6.1, 8.6)	–	–
Metformin/acarbose/glimepiride, *n* (%)	21 (61.8)	–	–
Insulin aspart/insulin glargine, *n* (%)	5 (14.7)	–	–
Acarbose and insulin glargine, *n* (%)	8 (23.5)	–	–

NDR: diabetic patients without diabetic retinopathy; BMI: body mass index; SBP: systolic blood pressure; DBP: diastolic blood pressure; TG: triglyceride; TC: total cholesterol; HDL: high-density lipoprotein; LDL: low-density lipoprotein; BCVA: best-corrected visual acuity; IOP: intraocular pressure; HbA1c: glycated haemoglobin.

### Retinal sensitivity and OCT measurements

Table 2 shows that the retinal sensitivity was lower in the NDR group than in the control group for all ETDRS sectors (all *p* <. 001). The p-RNFL of the NDR group was thinner in the T sector (76.24 ± 14.29 vs. 85.47 ± 19.66 µm, *p* = .035) but thicker in the N sector (77.41 ± 14.23 vs. 70.86 ± 15.40 µm, *p* = .029) than the control group. There were no differences in retinal thickness between the two groups in all ETDRS sectors, except for N2, where the NDR group had a thinner retina (304.55 ± 16.07 vs. 312.02 ± 12.30 µm, *p* = .010). There were no differences in retinal volume in all sectors (all *p* > .05, Supplement Table 2).

**Table 2.** Microperimetric retinal sensitivity (dB).

ETDRS sectors	NDR (*n* = 51)	Control (*n* = 49)	*p* ^a^
S	24.97 ± 1.14	26.40 ± 1.30	<.001
S1	25.38 ± 1.79	27.63 ± 1.19	<.001
S2	24.03 ± 1.32	25.53 ± 1.42	<.001
T	25.71 ± 1.31	27.49 ± 1.16	<.001
T1	26.34 ± 1.56	28.51 ± 1.19	<.001
T2	25.45 ± 1.32	26.72 ± 1.26	<.001
I	25.09 ± 1.00	26.62 ± 1.47	<.001
I1	26.15 ± 1.10	27.96 ± 1.33	<.001
I2	24.13 ± 1.36	25.80 ± 1.66	<.001
N	24.73 ± 1.26	26.33 ± 1.14	<.001
N1	26.37 ± 1.17	28.18 ± 1.28	<.001
N2	24.06 ± 1.09	25.26 ± 1.36	<.001
M	27.02 ± 1.17	29.07 ± 1.15	<.001

ETDRS: Early Treatment of Diabetic Retinopathy Study; NDR: diabetic patients without diabetic retinopathy; S: superior; T: temporal; I: inferior; N: nasal; M: middle.

^a^The *p*-values were adjusted for multiple comparisons using the Holm–Bonferroni method.

### OCTA measurements

The VD of RPCP was higher in the NDR group than in controls in the S, N, and I sectors (all *p* ≤ .001). Compared with the control group, PD of RPCP in the NDR group was lower in the T sector (96.78 ± 10.82 vs. 101.71 ± 15.40%, *p* = .030) but higher in the S (110.22 ± 7.33 vs. 105.51 ± 7.53%, *p* = .002) and N (101.53 ± 10.39 vs. 93.55 ± 10.42%, *p* < .001) sectors. The perimeter of the FAZ was larger in the NDR group compared with controls (2.63 ± 0.50 vs. 2.42 ± 0.39 mm, *p* = .026), without difference regarding area and circularity (both *p* > .05). There were no differences in VD of SCP between the two groups in all sectors (all *p* > .05), except for S1 and N1, which were lower in the NDR group than in controls (S1: 8.94 ± 0.90 vs. 9.90 ± 2.37%, *p* = .021; N1: 8.12 ± 1.42 vs. 9.53 ± 2.57%, *p* = .001). There were no differences in PD of SCP between the two groups in all sectors (all *p* > .05). There were no differences in VD and PD of DCP between the two groups in all sectors (all *p* > .05), except for N2 that PD was lower in the NDR group than in controls (44.92 ± 11.77 vs. 50.27 ± 6.37%, *p* = .044) (Table 3).

**Table 3.** Microvascular changes, VD and PD of RPCP, SCP, and DCP, and FAZ area, perimeter, and circularity.

Parameters	NDR (*n* = 51)	Control (*n* = 49)	*p*
Capillary drop-out, *n* (%)	40(78.4)	26(53.1)	.007
Notched or punched out borders, *n* (%)	44(86.3)	20(40.8)	<.001
Loss of smooth contour, *n* (%)	41(80.4)	13(26.5)	<.001
Cross vessel, *n* (%)	3(5.9)	0	.243*
VD of RPCP (%)			
T	21.41 ± 3.56	20.43 ± 2.95	.177#
S	22.20 ± 2.99	20.16 ± 2.27	<.001
N	21.20 ± 3.53	17.63 ± 2.33	<.001
I	20.98 ± 3.73	18.90 ± 2.31	.001
PD of RPCP (%)			
T	96.78 ± 10.82	101.71 ± 15.40	.030#
S	110.22 ± 7.33	105.51 ± 7.53	.002
N	101.53 ± 10.39	93.55 ± 10.42	<.001
I	103.61 ± 11.66	103.18 ± 9.88	.986#
FAZ			
Area (mm^2^)	0.34 ± 0.11	0.31 ± 0.10	.129
Perimeter (mm)	2.63 ± 0.50	2.42 ± 0.39	.026
Circularity	0.62 ± 0.10	0.65 ± 0.07	.077
VD of SCP (%)			
M	4.12 ± 1.48	3.76 ± 1.86	.237
S1	8.96 ± 0.89	9.92 ± 2.42	.030#
S2	9.69 ± 0.84	10.29 ± 2.13	.310#
T1	9.43 ± 1.02	10.11 ± 2.38	.078#
T2	8.92 ± 1.11	9.57 ± 1.88	.077#
I1	8.94 ± 1.29	9.60 ± 2.42	.331#
I2	9.80 ± 0.85	10.14 ± 2.23	.743#
N1	8.22 ± 1.47	9.49 ± 2.61	.006#
N2	10.55 ± 0.83	11.18 ± 2.42	.237#
PD of SCP (%)			
M	18.00 ± 7.19	19.53 ± 6.69	.274
S1	46.50 ± 3.80	44.96 ± 4.18	.056
S2	51.19 ± 4.09	52.12 ± 2.98	.150#
T1	47.56 ± 5.97	48.43 ± 5.27	.819#
T2	47.13 ± 5.70	48.29 ± 4.61	.461#
I1	45.04 ± 4.77	45.24 ± 5.63	.967#
I2	50.49 ± 5.43	52.20 ± 3.21	.331#
N1	43.13 ± 4.44	44.55 ± 4.64	.122
N2	52.36 ± 3.77	53.84 ± 2.12	.092#
VD of DCP (%)			
M	3.29 ± 1.77	3.04 ± 1.49	.629#
S1	10.80 ± 2.42	10.67 ± 2.42	.801#
S2	8.76 ± 2.31	8.22 ± 2.59	.281#
T1	10.84 ± 2.49	11.04 ± 2.35	.790#
T2	10.18 ± 2.74	10.14 ± 2.89	.925#
I1	9.82 ± 2.49	9.71 ± 2.67	.746#
I2	7.55 ± 2.65	7.33 ± 2.55	.600#
N1	11.10 ± 2.19	11.10 ± 2.39	.994#
N2	9.84 ± 2.39	9.39 ± 2.27	.169#
PD of DCP (%)			
M	15.76 ± 9.48	14.75 ± 6.67	.986#
S1	48.47 ± 11.43	47.43 ± 9.78	.481#
S2	38.55 ± 11.12	39.22 ± 9.08	.904#
T1	49.06 ± 12.33	49.82 ± 10.68	.915#
T2	45.22 ± 12.24	46.29 ± 10.89	.775#
I1	43.27 ± 12.42	44.33 ± 11.03	.893#
I2	33.98 ± 11.95	36.12 ± 10.11	.492#
N1	49.55 ± 12.51	50.59 ± 9.81	.964#
N2	44.92 ± 11.77	50.27 ± 6.37	.044#

#At least one group of the parameter did not conform to the normal distribution, and the *p*-value was obtained by the Mann–Whitney *U* test.

*Fisher exact test.

VD: vascular density; PD: perfusion density; RPCP: radial peripapillary capillary plexus; SCP: superficial capillary plexus; DCP: deep capillary plexus; FAZ: foveal avascular zone; NDR: diabetic patients without diabetic retinopathy; T: temporal; S: superior; N: nasal; I: inferior; M: middle.

In the NDR group, 78.4% of the patients (40 of 51 eyes) had perifoveal capillary drop-out, 86.3% of the patients (44 of 51 eyes) had notched or punched out borders of the superficial FAZ, 80.4% of the patients (41 of 51 eyes) demonstrated loss of smooth contour, and three patient demonstrated vessels crossing through the fovea. In the control group, 53.1% of the patients (26 of 49 eyes) had perifoveal capillary drop-out, 40.8% of patients (20 of 49 eyes) had notched or punched out borders of the superficial FAZ, 26.5% of patients (13 of 49 eyes) demonstrated loss of smooth contour, and none of the patients demonstrated vessels crossing through the fovea. The frequencies of perifoveal capillary drop-out, notched or punched out borders of the superficial FAZ, and loss of smooth contour were all higher in the NDR group (all *p* < .05, Table 3).

## Correlations

Table 4 shows that retinal volume S2 was correlated with SCP PD S2 (*r* = 0.371, *p* < .01); retinal volume M was correlated with SCP PD M (*r* = 0.415, *p* < .01), DCP VD M (*r* = 0.503, *p* < .01), and DCP PD M (*r* = 0.519, *p* < .01); retinal thickness S2 was correlated with SCP PD S2 (*r* = 0.549, *p* < .01); retinal thickness M was correlated with DCP VD M (*r* = 0.527, *p* < .01) and DCP PD M (*r* = 0.526, *p* < .01) in the NDR group. The retinal sensitivity was only correlated with PD of SCP in T2 sector (*r* = 0.262, *p* < .01) and VD of SCP in T1 sector (*r* = 0.224, *p* < .05) (Supplement Table 3).

**Table 4.** Correlation of retinal volume, retinal thickness, and VD and PD of SCP and VD and PD of DCP in the NDR group.

	SPD M	SPD S2	DVD M	DPD M
Retinal volume				
Retinal volume S2		0.371**		
Retinal volume M	0.415**		0.503**	0.519**
Retinal thickness				
Retinal thickness S2		0.549**		
Retinal thickness M			0.527**	0.526**

**p < .01.

SPD: perfusion density of superficial capillary plexus; DVD: vessel density of deep capillary plexus; DPD: perfusion density of deep capillary plexus M: middle S: superior.

## Discussion

The characteristics of the early changes in preclinical DR are poorly known. DR is diagnosed in the presence of microaneurysms, but other changes can occur before overt DR. This study aimed to analyse the changes in the structure and function of the fundus in NDR. The results suggest that the structure (p-RNFL thickness, VD, and PD) and function (retinal sensitivity) display some changes in NDR.

The MP-3 microperimeter is a visual function inspection device developed in recent years based on the laser scanning ophthalmoscope technique. It is non-invasive, fast, with accurate and reliable results, and can quantitatively evaluate the macular visual sensitivity and position and stability of fixation. It can detect the changes in retinal sensitivity in patients with early-stage DM and help reduce the false-negative rate of conventional examining methods. In addition, the retinal sensitivity results can be matched with images of the fundus photochromy so that the functional changes of the retina can accurately correspond to specific structures of the fundus. In the present study, the patients with NDR showed decreased retinal sensitivity in all sectors of the retina. Verma et al. [23] and Neriyanuri et al. [24] used microperimetry, as in the present study, and showed decreased retinal sensitivity in NDR and suggested that this decrease could be an early sign of DR. Such changes can be due to neurodegeneration, hypoxia, and oxidative stress [24]. Abnormal function often suggests histomorphological changes. However, in the present study, the NDR group had a thinner retina only in N2, and the retinal thickness in other sectors was not statistically different from the control group. There was no significant correlation between retinal sensitivity and retinal thickness in all sectors of the ETDRS chart. In addition, retinal sensitivity showed no significant correlation with VD and PD in SCP, except for PD in T2 and VD in T1 sectors, respectively, which were only weakly correlated.

DR is a combination of microvascular abnormalities and neurodegenerative changes in the retinal ganglion cells [25,26]. Previous studies showed that the mean four-quadrant p-RNFL was thinner in NDR patients than in controls [27,28]. Sohn et al. [29] also showed that these changes were independent of age, sex, and glycemic control. Still, the present study showed that p-RNFL was thinner in the T sector but thicker in the N sector, which probably indicated that the RNFL was thicker but to the detriment of another layer. Lopes de Faria et al. [30] also showed that the p-RNFL was thinner in the superior quadrants in NDR. The exact reasons for the different changes in different parts of the retina are currently unknown, as supported by Dhasmana et al. [31]. Nevertheless, the present and previous studies indicate some degree of retinal neurodegeneration before the development of overt DR.

In this study, the patients with NDR showed higher frequencies of perifoveal capillary drop-out, notched or punched out borders of the superficial FAZ, and loss of smooth contour compared with the control group. These changes are consistent with the literature [32,33].

Previous studies reported a larger FAZ area in NDR than in healthy controls [34–37]. Tang et al. [34] also mentioned that the severity of DR was associated with the enlargement of the FAZ. In the present study, the NDR group had a similar FAZ area to the control group, supported by Mastropasqua et al. [38]. The inconsistency of these results might be due to the high interindividual variation of the FAZ surface [39].

This study showed differences in VD and PD between NDR and controls, but only in specific sectors of the retina. Yang et al. [5] showed that NDR had lower VD in the foveal region of the superficial retinal layer but higher VD in the parafoveal region of the deep layer. These results were supported by other studies [35,40]. Notably, PD of DCP and retinal thickness were lower in the N2 sector in the NDR group than in controls. There might be a relationship between a thinner retina and decreased PD, but further studies are needed to verify it. Again, the reasons why only specific sectors of the retina are affected are unknown and will require additional studies.

The present study showed that PD and thickness in the T region of RPCP and p-RNFL were decreased in the NDR group compared with the control group. It is supported by Shin et al. [41], who showed that the peripapillary VD and PD in SCP were correlated with the p-RNFL thickness. These changes are likely due to the loss of astrocytes and ganglion cells in diabetes [41]. The RNFL contains the axons ganglion cells towards the optic disc, and a reduced RNFL thickness might be related to decreased microvascular density. Previous studies reported that microvascular abnormalities in diabetic patients were not uniformly distributed in each quadrant of the retina, and the abnormalities were more common in the superior temporal quadrant than in the inferior nasal quadrant of the retina [42,43]. In addition, the present study showed that VD and PD of certain sectors were higher in the NDR group than in the controls. However, this was inconsistent with a previous meta-analysis which showed reduced PD in RPCP [44]. The possible reasons for these results are unexpected and need further exploration. Notably, in the macular sector N2, the retinal thickness and PD of DCP in the NDR group were also lower than in the control group. Both the T sector of RPCP and N2 sector of the macula are located in the papilomacular area, suggesting that lesions of the papillomacular bundle were more obvious in diabetic patients prior to the onset of clinically significant retinopathy. Based on the correlation analysis of retinal thickness, volume, VD, and PD in the patients with NDR, it can be seen that, in the stage of non-diabetic retinopathy, there is a certain correlation between the microvascular changes and the changes in retinal thickness and volume, and most of them occur in area M. A possible cause might be that in the pathogenesis of DR, endothelial dysfunction and increased leukocyte and ICAM-1 expression are involved in the destruction of the vascular network structure, leading to a series of morphological changes in the FAZ region [45–47]. Additional studies are necessary to determine the exact pathogenesis events.

In addition, Srinivasan et al. [48] showed that diabetic patients with NDR display subclinical changes in retinal and visual function parameters, which were associated with subclinical retinal structural changes. Nevertheless, the cause is still unclear, and large sample studies are needed for confirmation. Whether neurodegeneration or microangiopathy comes first before retinopathy in diabetic patients is still unknown. Since diabetes is a disease with neurovascular implications, this study was conducted from both the functional and structural aspects. Although this study found that the retinal structure and function of diabetes patients without DR have different degrees of change, it is still not possible to prove their relationship in time.

This study has limitations. It was a single-centre study with a small sample size. The data collection was cross-sectorial, preventing the analysis of any causal relationship. Due to the exclusion criteria, not all individuals had two eligible eyes, which could bias the analyses. Future studies should be longitudinal to observe the dynamic changes in structure and function during the development of DR.

In conclusion, the structure (p-RNFL thickness, VD, PD) and function (retinal sensitivity) display some changes in diabetic patients even before the overt development of DR.

## Supplementary Material

Supplemental Material

## Data Availability

The datasets used and/or analysed during the current study are available from the corresponding author on reasonable request.

## References

[1] American Academy of Ophthalmology. Diabetic retinopathy preferred practice pattern – updated 2019. San Francisco: American Academy of Ophthalmology; 2019.

[2] Yau JWY, Rogers SL, Kawasaki R, Meta-Analysis for Eye Disease (META-EYE) Study Group, et al. Global prevalence and major risk factors of diabetic retinopathy. Diabetes Care. 2012;35(3):556–564. 22301125 10.2337/dc11-1909 PMC3322721

[3] Wong TY, Cheung CMG, Larsen M, et al. Diabetic retinopathy. Nat Rev Dis Primers. 2016;2:16012. 27159554 10.1038/nrdp.2016.12

[4] American Diabetes Association. 11. Microvascular complications and foot care: standards of medical care in diabetes-2021. Diabetes Care. 2021;44(Suppl 1):S151–S167. 33298422 10.2337/dc21-S011

[5] Yang JY, Wang Q, Yan YN, et al. Microvascular retinal changes in preclinical diabetic retinopathy as detected by optical coherence tomographic angiography. Graefes Arch Clin Exp Ophthalmol. 2020;258(3):513–520. 31897704 10.1007/s00417-019-04590-x

[6] Chen X, Nie C, Gong Y, et al. Peripapillary retinal nerve fiber layer changes in preclinical diabetic retinopathy: a meta-analysis. PLOS One. 2015;10(5):e0125919. 25965421 10.1371/journal.pone.0125919 PMC4429076

[7] Gonul S, et al. Evaluation of retinal nerve fiber layer thickness with optical coherence tomography in type 1 diabetes mellitus patients. Turkiye Klinikleri J Med Sci. 2011;31:1100–1105.

[8] Peng PH, Lin HS, Lin S. Nerve fibre layer thinning in patients with preclinical retinopathy. Can J Ophthalmol. 2009;44(4):417–422. 19606163 10.3129/i09-112

[9] Park HY, Kim IT, Park CK. Early diabetic changes in the nerve fibre layer at the macula detected by spectral domain optical coherence tomography. Br J Ophthalmol. 2011;95(9):1223–1228. 21216799 10.1136/bjo.2010.191841

[10] Sugimoto M, Sasoh M, Ido M, et al. Detection of early diabetic change with optical coherence tomography in type 2 diabetes mellitus patients without retinopathy. Ophthalmologica. 2005;219(6):379–385. 16286799 10.1159/000088382

[11] Lung JCY, Swann PG, Wong DSH, et al. Global flash multifocal electroretinogram: early detection of local functional changes and its correlations with optical coherence tomography and visual field tests in diabetic eyes. Doc Ophthalmol. 2012;125(2):123–135. 22828871 10.1007/s10633-012-9343-0

[12] Xin C, Wang J, Meng X, et al. Effect on the retinal fiber thickness in early diabetes. Zhonghua Yi Xue Za Zhi. 2014;94(3):208–211. 24731465

[13] Ma J, et al. Correlation of optic retinal nerve fiber layer thickness and visual function in patients with nonproliferative diabetic retinopathy. Zhonghua Yan Ke Za Zhi. 2013;49(6):514–520. 24119964

[14] Oshitari T, Hanawa K, Adachi-Usami E. Changes of macular and RNFL thicknesses measured by stratus OCT in patients with early stage diabetes. Eye. 2009;23(4):884–889. 18437178 10.1038/eye.2008.119

[15] Li Z, Wen X, Zeng P, et al. Do microvascular changes occur preceding neural impairment in early-stage diabetic retinopathy? Evidence based on the optic nerve head using optical coherence tomography angiography. Acta Diabetol. 2019;56(5):531–539. 30656435 10.1007/s00592-019-01288-8

[16] Kim K, Kim ES, Kim DG, et al. Progressive retinal neurodegeneration and microvascular change in diabetic retinopathy: longitudinal study using OCT angiography. Acta Diabetol. 2019;56(12):1275–1282. 31401734 10.1007/s00592-019-01395-6

[17] Pires I, Bernardes RC, Lobo CL, et al. Retinal thickness in eyes with mild nonproliferative retinopathy in patients with type 2 diabetes mellitus: comparison of measurements obtained by retinal thickness analysis and optical coherence tomography. Arch Ophthalmol. 2002;120(10):1301–1306. 12365908 10.1001/archopht.120.10.1301

[18] Park JC, Chen Y-F, Liu M, et al. Structural and functional abnormalities in early-stage diabetic retinopathy. Curr Eye Res. 2020;45(8):975–985. 31847599 10.1080/02713683.2019.1705983 PMC7347436

[19] Gella L, Raman R, Kulothungan V, et al. Retinal sensitivity in subjects with type 2 diabetes mellitus: Sankara nethralaya diabetic retinopathy epidemiology and molecular genetics study (SN-DREAMS II, report No. 4). Br J Ophthalmol. 2016;100(6):808–813. 26338972 10.1136/bjophthalmol-2015-307064

[20] Zeng Y, et al. Early retinal neurovascular impairment in patients with diabetes without clinically detectable retinopathy. Br J Ophthalmol. 2019;103(12):1747–1752. 30674454 10.1136/bjophthalmol-2018-313582

[21] Wilkinson CP, Ferris FL, Klein RE, Global Diabetic Retinopathy Project Group, et al. Proposed international clinical diabetic retinopathy and diabetic macular edema disease severity scales. Ophthalmology. 2003;110(9):1677–1682. 13129861 10.1016/S0161-6420(03)00475-5

[22] Palkovits S, Hirnschall N, Georgiev S, et al. Effect of cataract extraction on retinal sensitivity measurements. Ophthalmic Res. 2021;64(1):10–14. 32209789 10.1159/000507450

[23] Verma A, Rani PK, Raman R, et al. Is neuronal dysfunction an early sign of diabetic retinopathy? Microperimetry and spectral domain optical coherence tomography (SD-OCT) study in individuals with diabetes, but no diabetic retinopathy. Eye. 2009;23(9):1824–1830. 19648899 10.1038/eye.2009.184

[24] Neriyanuri S, Pardhan S, Gella L, et al. Retinal sensitivity changes associated with diabetic neuropathy in the absence of diabetic retinopathy. Br J Ophthalmol. 2017;101(9):1174–1178. 28108570 10.1136/bjophthalmol-2016-309641

[25] Altmann C, Schmidt MHH. The role of microglia in diabetic retinopathy: inflammation, microvasculature defects and neurodegeneration. Int J Mol Sci. 2018;19(1):110. 10.3390/ijms19010110 PMC5796059 29301251

[26] Barber AJ. A new view of diabetic retinopathy: a neurodegenerative disease of the eye. Prog Neuropsychopharmacol Biol Psychiatry. 2003;27(2):283–290. 12657367 10.1016/S0278-5846(03)00023-X

[27] Mehboob MA, Amin ZA, Islam QU. Comparison of retinal nerve fiber layer thickness between normal population and patients with diabetes mellitus using optical coherence tomography. Pak J Med Sci. 2019;35(1):29–33. 30881391 10.12669/pjms.35.1.65 PMC6408645

[28] Karti O, Nalbantoglu O, Abali S, et al. Retinal ganglion cell loss in children with type 1 diabetes mellitus without diabetic retinopathy. Ophthalmic Surg Lasers Imaging Retina. 2017;48(6):473–477. 28613353 10.3928/23258160-20170601-05

[29] Sohn EH, van Dijk HW, Jiao C, et al. Retinal neurodegeneration may precede microvascular changes characteristic of diabetic retinopathy in diabetes mellitus. Proc Natl Acad Sci USA. 2016;113(19):E2655–64. 27114552 10.1073/pnas.1522014113 PMC4868487

[30] Lopes de Faria JM, Russ H, Costa VP. Retinal nerve fibre layer loss in patients with type 1 diabetes mellitus without retinopathy. Br J Ophthalmol. 2002;86(7):725–728. 12084737 10.1136/bjo.86.7.725 PMC1771182

[31] Dhasmana R, Sah S, Gupta N. Study of retinal nerve fibre layer thickness in patients with diabetes mellitus using Fourier domain optical coherence tomography. J Clin Diagn Res. 2016;10(7):NC05–9. 10.7860/JCDR/2016/19097.8107 PMC5020191 27630874

[32] Oliverio GW, Ceravolo I, Bhatti A, et al. Foveal avascular zone analysis by optical coherence tomography angiography in patients with type 1 and 2 diabetes and without clinical signs of diabetic retinopathy. Int Ophthalmol. 2021;41(2):649–658. 33156947 10.1007/s10792-020-01621-z

[33] Fleissig E, Adhi M, Sigford DK, et al. Foveal vasculature changes and nonperfusion in patients with diabetes types I and II with no evidence of diabetic retinopathy. Graefes Arch Clin Exp Ophthalmol. 2020;258(3):551–556. 31900642 10.1007/s00417-019-04588-5

[34] Tang FY, Ng DS, Lam A, et al. Determinants of quantitative optical coherence tomography angiography metrics in patients with diabetes. Sci Rep. 2017;7(1):2575. 28566760 10.1038/s41598-017-02767-0 PMC5451475

[35] Durbin MK, An L, Shemonski ND, et al. Quantification of retinal microvascular density in optical coherence tomographic angiography images in diabetic retinopathy. JAMA Ophthalmol. 2017;135(4):370–376. 28301651 10.1001/jamaophthalmol.2017.0080 PMC5470403

[36] Takase N, Nozaki M, Kato A, et al. Enlargement of foveal avascular zone in diabetic eyes evaluated by en face optical coherence tomography angiography. Retina. 2015;35(11):2377–2383. 26457396 10.1097/IAE.0000000000000849

[37] Lee J, Moon BG, Cho AR, et al. Optical coherence tomography angiography of DME and its association with anti-VEGF treatment response. Ophthalmology. 2016;123(11):2368–2375. 27613201 10.1016/j.ophtha.2016.07.010

[38] Mastropasqua R, et al. Foveal avascular zone area and parafoveal vessel density measurements in different stages of diabetic retinopathy by optical coherence tomography angiography. Int J Ophthalmol. 2017;10(10):1545–1551. 29062774 10.18240/ijo.2017.10.11 PMC5638976

[39] Lupidi M, Coscas G, Coscas F, et al. Retinal microvasculature in nonproliferative diabetic retinopathy: Automated quantitative optical coherence tomography angiography assessment. Ophthalmic Res. 2017;58(3):131–141. 28538221 10.1159/000471885

[40] Simonett JM, Scarinci F, Picconi F, et al. Early microvascular retinal changes in optical coherence tomography angiography in patients with type 1 diabetes mellitus. Acta Ophthalmol. 2017;95(8):e751–e755. 28211261 10.1111/aos.13404

[41] Shin Y-I, Nam KY, Lee SE, et al. Peripapillary microvasculature in patients with diabetes mellitus: an optical coherence tomography angiography study. Sci Rep. 2019;9(1):15814. 31676848 10.1038/s41598-019-52354-8 PMC6825207

[42] Robinson R, Barathi VA, Chaurasia SS, et al. Update on animal models of diabetic retinopathy: from molecular approaches to mice and higher mammals. Dis Model Mech. 2012;5(4):444–456. 22730475 10.1242/dmm.009597 PMC3380708

[43] Kim JS. The microperimetry of resolved cotton-wool spots in eyes of patients with hypertension and diabetes mellitus. Arch Ophthalmol. 2011;129(7):879–884. 21746978 10.1001/archophthalmol.2011.51 PMC13068502

[44] Zhang B, Chou Y, Zhao X, et al. Early detection of microvascular impairments with optical coherence tomography angiography in diabetic patients without clinical retinopathy: a meta-analysis. Am J Ophthalmol. 2021;222:226–237. 32976846 10.1016/j.ajo.2020.09.032

[45] Cade WT. Diabetes-related microvascular and macrovascular diseases in the physical therapy setting. Phys Ther. 2008;88(11):1322–1335. 18801863 10.2522/ptj.20080008 PMC2579903

[46] Matsunaga DR, Yi JJ, De Koo LO, et al. Optical coherence tomography angiography of diabetic retinopathy in human subjects. Ophthalmic Surg Lasers Imaging Retina. 2015;46(8):796–805. 26431294 10.3928/23258160-20150909-03

[47] Miyamoto K, Khosrof S, Bursell SE, et al. Prevention of leukostasis and vascular leakage in streptozotocin-induced diabetic retinopathy via intercellular adhesion molecule-1 inhibition. Proc Natl Acad Sci USA. 1999;96(19):10836–10841. 10485912 10.1073/pnas.96.19.10836 PMC17969

[48] Srinivasan S, Rajalakshmi R, Anjana RM, et al. Retinal structure-function correlation in type 2 diabetes. Eye(Lond). 2021. 10.1038/s41433-021-01761-1 PMC9500073 34462581

