# Supplementary material for: Structural and functional retinal changes in patients with type 2 diabetes without diabetic retinopathy
Source: Ann Med. 2022 Jul 4;54(1):1816–25. doi: 10.1080/07853890.2022.2095010 (PMC9258434; doi:10.1080/07853890.2022.2095010)
Supplement: Supplemental Material [file IANN_A_2095010_SM2091.docx]

**Supplement Table 1** Exclusion criteria of two groups

| **Control group** | |
| --- | --- |
| **One eye is normal，the other eye has the following conditions to be ruled out** | **N (eyes)** |
| 1. Diopter ≥+3.00 D or ≤-3.00 D | 5 (5) |
| 2. Vitreous hemorrhage caused by acute posterior vitreous detachment | 2 (2) |
| 3. Branch retinal vein occlusion without any other systemic disease | 3 (3) |
| 4. Idiopathic epiretinal membrane | 2 (2) |
| 5.Severe lens opacity that affects the fundus examination | 7 (7) |
| 6.Fail to obtain qualified images or incomplete data | 6 (6) |
| **Both eyes are normal** | 12 (24) |
| **Total** | 37 (49) |
| **NDR group** | |
| 1. Fail to obtain qualified images or incomplete data | 7 (7) |
| 5. Severe lens opacity that affects the fundus examination | 6 (6) |
| 3. Proliferative diabetic retinopathy | 4 (4) |
| **Both eyes are included** | 17 (34) |
| **Total** | 34 (51) |

**Supplement Table 2.** P-RNFL thickness and macular retinal thickness, and retinal volume

| Parameters | NDR (n=51) | Control (n=49) | P |
| --- | --- | --- | --- |
| p-RNFL thickness (μm) |  |  |  |
| T | 76.24±14.29 | 85.47±19.66 | 0.035 # |
| S | 136.65±18.75 | 139.76±16.95 | 0.387 |
| N | 77.41±14.23 | 70.86±15.40 | 0.029 |
| I | 144.71±17.70 | 139.76±13.36 | 0.119 |
| Retinal thickness (μm) |  |  |  |
| M | 249.12±16.06 | 253.37±15.54 | 0.182 |
| S1 | 330.57±18.18 | 334.51±12.96 | 0.214 |
| S2 | 295.04±15.98 | 297.22±11.78 | 0.437 |
| T1 | 318.90±18.03 | 320.80±11.93 | 0.536 |
| T2 | 285.51±23.69 | 282.16±9.68 | 0.850 # |
| I1 | 328.16±17.09 | 331.02±13.16 | 0.352 |
| I2 | 281.20±13.54 | 282.39±10.68 | 0.692 # |
| N1 | 329.84±17.34 | 334.22±14.02 | 0.169 |
| N2 | 304.55±16.07 | 312.02±12.298 | 0.010 |
| Retinal volume (mm^3^) |  |  |  |
| M | 0.20±0.01 | 0.20±0.01 | 0.335 # |
| S1 | 0.52±0.03 | 0.53±0.02 | 0.292 |
| S2 | 1.58±0.07 | 1.58±0.06 | 0.661 |
| T1 | 0.50±0.03 | 0.51±0.02 | 0.350 |
| T2 | 1.52±0.10 | 1.50±0.05 | 0.607 # |
| I1 | 0.52±0.03 | 0.52±0.02 | 0.320 |
| I2 | 1.50±0.06 | 1.50±0.06 | 0.472 # |
| N1 | 0.52±0.03 | 0.53±0.02 | 0.330 |
| N2 | 1.63±0.08 | 1.65±0.07 | 0.096 |

# At least one group of the parameter did not conform to the normal distribution, and the P-value was obtained by the Mann-Whitney U test.

NDR: diabetic patients without diabetic retinopathy; p-RNFL: peripapillary retinal nerve fiber layer; T: temporal; S: superior; N: nasal; I: inferior; M: middle.

**Supplement Table 3.** Correlation of retinal sensitivity with VD and PD of SCP, retinal thickness, and retinal volume (n=100).

|  | S1 | S2 | T1 | T2 | I1 | I2 | N1 | N2 |
| --- | --- | --- | --- | --- | --- | --- | --- | --- |
| SCP-PD S1 | -0.072 |  |  |  |  |  |  |  |
| SCP-PD S2 |  | 0.120 |  |  |  |  |  |  |
| SCP-PD T1 |  |  | 0.080 |  |  |  |  |  |
| SCP-PD T2 |  |  |  | 0.262** |  |  |  |  |
| SCP-PD I1 |  |  |  |  | -0.082 |  |  |  |
| SCP-PD I2 |  |  |  |  |  | 0.077 |  |  |
| SCP-PD N1 |  |  |  |  |  |  | -0.099 |  |
| SCP-PD N2 |  |  |  |  |  |  |  | 0.044 |
| SCP-VD S1 | 0.148 |  |  |  |  |  |  |  |
| SCP-VD S2 |  | -0.009 |  |  |  |  |  |  |
| SCP-VD T1 |  |  | 0.224* |  |  |  |  |  |
| SCP-VD T2 |  |  |  | 0.167 |  |  |  |  |
| SCP-VD I1 |  |  |  |  | 0.051 |  |  |  |
| SCP-VD I2 |  |  |  |  |  | 0.087 |  |  |
| SCP-VD N1 |  |  |  |  |  |  | 0.154 |  |
| SCP-VD N2 |  |  |  |  |  |  |  | 0.053 |
| Retinal thickness S1 | -0.009 |  |  |  |  |  |  |  |
| Retinal thickness S2 |  | -0.015 |  |  |  |  |  |  |
| Retinal thickness T1 |  |  | -0.171 |  |  |  |  |  |
| Retinal thickness T2 |  |  |  | 0.154 |  |  |  |  |
| Retinal thickness I1 |  |  |  |  | -0.066 |  |  |  |
| Retinal thickness I2 |  |  |  |  |  | 0.008 |  |  |
| Retinal thickness N1 |  |  |  |  |  |  | -0.070 |  |
| Retinal thickness N2 |  |  |  |  |  |  |  | 0.049 |
| Retinal volume S1 | -0.043 |  |  |  |  |  |  |  |
| Retinal volume S2 |  | -0.095 |  |  |  |  |  |  |
| Retinal volume T1 |  |  | -0.183 |  |  |  |  |  |
| Retinal volume T2 |  |  |  | 0.109 |  |  |  |  |
| Retinal volume I1 |  |  |  |  | -0.064 |  |  |  |
| Retinal volume I2 |  |  |  |  |  | -0.019 |  |  |
| Retinal volume N1 |  |  |  |  |  |  | -0.108 |  |
| Retinal volume N2 |  |  |  |  |  |  |  | -0.030 |

*P<0.05, **P<0.01.

SCP: superficial capillary plexus; PD: perfusion density; VD: vascular density; S: superior; T: temporal; I: inferior; N: nasal.
